# An evidence mapping study based on systematic reviews of traditional Chinese medicine for hyperuricemia

**DOI:** 10.3389/fnut.2026.1811252

**Published:** 2026-04-23

**Authors:** Jiahui Li, Ziqi Cao, Shien Cui, Wenzheng Tang, Xinyu Gao, Yingshuai Li

**Affiliations:** 1 School of Traditional Chinese Medicine, Beijing University of Chinese Medicine, Beijing, China; 2 National Institute of Traditional Chinese Medicine Constitution and Preventive Treatment of Diseases, Beijing University of Chinese Medicine, Beijing, China

**Keywords:** evidence mapping, hyperuricemia, meta-analysis, systematic review, TCM

## Abstract

**Objective:**

This study aimed to comprehensively evaluate the methodological quality, reporting quality, and certainty of evidence of systematic reviews (SRs) on Traditional Chinese Medicine (TCM) for hyperuricemia (HUA) using an umbrella review integrated with evidence mapping.

**Methods:**

Systematic searches were performed in PubMed, The Cochrane Library, Embase, Web of Science (WOS), CNKI, WanFang, SinoMed, and VIP to identify SRs evaluating TCM for HUA published up to December 2025. Methodological quality was assessed using the AMSTAR 2 tool, reporting completeness was evaluated according to the PRISMA statement, and the certainty of evidence for outcome measures was graded using the GRADE system.

**Results:**

Eleven SRs were ultimately included in the analysis. Eight SRs evaluated the total effective rate, demonstrating that both TCM monotherapy and integrated Traditional Chinese and Western medicine (ITCW) treatments were superior to Western medicine (WM) alone. Ten SRs assessed serum uric acid (sUA) levels, showing that both TCM and ITCW regimens outperformed WM or placebo. Six SRs analyzed adverse reaction incidence, demonstrating that the application of TCM and ITCW was associated with a lower incidence of adverse events compared with WM. The AMSTAR 2 assessment revealed that the methodological quality of all included studies was rated as critically low, with primary deficiencies being the lack of protocol registration and the failure to provide a list of excluded studies. The PRISMA evaluations showed a polarization in reporting quality, with severe deficiencies in key items such as protocol registration, funding disclosure, and conflict of interest reporting. The GRADE assessments indicated that the certainty of evidence for the majority of outcome measures was low or very low, primarily downgraded due to study limitations and inconsistency.

**Conclusion:**

Although TCM and ITCW interventions showed potential benefits for favorable efficacy and safety in managing hyperuricemia, the pooled results are derived from studies of critically low methodological quality and therefore do not support definitive clinical recommendations. Existing systematic reviews suffer from poor methodological rigor and suboptimal reporting, highlighting an urgent need for standardized reporting guidelines. Future research should emphasize rigorous study design and mandatory protocol registration to generate high-quality evidence from well-conducted randomized controlled trials (RCTs).

## 1 Introduction

Hyperuricemia (HUA), a metabolic disorder characterized by serum uric acid levels exceeding 7 mg/dL in male patients and 6 mg/dL in female patients, stems from enhanced uric acid synthesis or diminished excretion. This condition is strongly associated with a spectrum of severe complications, including gout, renal impairment, cardiovascular diseases, and diabetes, thereby positioning it as a critical global public health concern (1). In China, the prevalence of HUA is increasing annually and affecting younger populations, substantially compromising patients’ quality of life and increasing healthcare expenditure (2). Although conventional therapies rely on medications such as allopurinol and febuxostat, their clinical utility is often hampered by adverse events such as hepatorenal dysfunction and gastrointestinal distress, hindering effective long-term comprehensive management. In contrast, TCM adopts a holistic philosophical framework, categorizing HUA within the early stages of “Li Jie” and “Bi Zheng,” viewing its pathogenesis as a combination of root deficiency and tip excess. TCM employs treatment based on pattern differentiation using single herbs or complex formulas to regulate uric acid metabolism. These methods offer advantages such as multi-target efficacy and fewer side effects, leading to widespread clinical adoption (3). Increasing evidence demonstrates that TCM agents—including the single herbs *Reynoutria japonica* (Huzhang) (4) and *Plantago asiatica* (Cheqiancao) (5), as well as formulas such as Simiao Pill (6) and Guizhi Shaoyao Zhimu Decoction (7)—exert therapeutic effects through mechanisms such as inhibiting xanthine oxidase activity, regulating uric acid transporters (e.g., URAT1), and ameliorating inflammation responses. In contrast, external TCM therapies and physiotherapies exhibit substantial clinical heterogeneity regarding formulation choices, application sites, and operational protocols. Furthermore, high-quality original research supporting these modalities remains relatively scarce. Therefore, this study exclusively focuses on oral TCM treatments for HUA.

Although several SRs have explored TCM for HUA, these studies are limited by variable evidence quality, high methodological heterogeneity, and contradictory conclusions. Consequently, a comprehensive synthesis and visualization of the existing evidence remains lacking. The umbrella review methodology enables a multi-level synthesis and structured organization of related SRs, enabling the standardized assessment of efficacy, safety, and evidence certainty (8). This approach effectively addresses the challenges of dispersed findings and prominent heterogeneity inherent in TCM research, thereby facilitating the comparability of results. Furthermore, evidence mapping systematically identifies evidence distribution and research gaps across various interventions and outcome measures, fitting the paradigm of TCM’s pattern differentiation and multi-dimensional treatment strategies.

Therefore, this study employs an umbrella systematic review integrated with evidence mapping to comprehensively identify SRs related to TCM for HUA. Our objectives are to systematically assess the overall quality, consistency of efficacy, and safety of the current evidence base and to map the distribution of evidence, highlighting both research strengths and critical gaps. Ultimately, this study aims to synthesize available data to provide an evidence-based foundation for the standardized clinical application of TCM in HUA, while also identifying research gaps to guide future clinical trial design and mechanistic investigations, thereby advancing the evidence-based development of TCM in the field of metabolic disorders.

## 2 Materials and methods

### 2.1 Inclusion and exclusion criteria

#### 2.1.1 Study type

This study conducts a secondary analysis of the included SRs, rather than performing a systematic review of the original RCTs. The scope was limited to studies published in Chinese or English.

#### 2.1.2 Study subjects (population)

Patients diagnosed with hyperuricemia, including asymptomatic hyperuricemia and gout in remission with hyperuricemia, were included. Patients with diabetes mellitus and other metabolic disorders were excluded.

#### 2.1.3 Interventions and comparisons

The intervention group received Traditional Chinese Medicine monotherapy or integrated Traditional Chinese and Western medicine treatment. The control group received conventional Western medicine therapy, placebo, or no intervention.

#### 2.1.4 Outcome measures

Outcome measures included, but were not limited to, serum uric acid levels, total efficacy rate, and the incidence of adverse reactions, as reported in all included studies.

#### 2.1.5 Exclusion criteria

The exclusion criteria were as follows: (A) systematic reviews or meta-analyses synthesizing non-randomized controlled trials (non-RCTs); (B) insufficient data regarding primary outcome measures or other essential indicators, precluding effective data extraction; (C) interventions employing external therapy of TCM, such as acupoint application, acupoint injection, acupuncture, or Tui Na massage; (D) studies involving patients whose hyperuricemia was complicated by concurrent chronic conditions, such as diabetes, to isolate the baseline efficacy of TCM and avoid complex metabolic confounders; and (E) duplicate publications.

### 2.2 Search strategy

A comprehensive literature search was conducted across multiple English and Chinese academic databases to identify relevant SRs focusing on TCM for HUA. Specifically, the English databases included PubMed, The Cochrane Library, Embase, and WOS, while the Chinese databases included CNKI, WanFang, SinoMed, and VIP. The search timeframe spanned from the inception of each respective database to December 2025. All searches were performed using a combined strategy of Medical Subject Headings (MeSH) terms and free-text keywords to ensure comprehensive retrieval. The core English search terms were as follows: Traditional Chinese Medicine, Chinese herbal medicine, Hyperuricemia, and meta-analysis. Detailed search protocols for each individual database are documented in Supplementary Table S1.

Literature screening and data extraction were conducted independently by two trained reviewers, who initially screened all retrieved records by reviewing titles and abstracts, followed by a cross-verification process to ensure consistency. Studies that failed to meet the predefined eligibility criteria were excluded during the initial screening phase. Subsequently, the full texts of the remaining potentially eligible studies were thoroughly evaluated to determine the final set of the included cohort of systematic reviews. Any disagreements or discrepancies between the two independent reviewers were resolved through consensus, following consultation with a third senior reviewer. The following core data were systematically extracted: (A) basic study characteristics, including authors, publication year, specific interventions in the experimental and control groups, quality assessment instruments, and defined outcome measures; (B) key methodological features of the included systematic reviews; and (C) pooled statistical analysis results and relevant outcome data.

### 2.3 Evaluation methods

Methodological quality and reporting completeness of the included SRs were independently appraised by two reviewers against standardized assessment tools, with any inter-reviewer discrepancies resolved through consensus, involving consultation with a third independent reviewer when necessary. Statistical analysis was performed using R statistical software (version 4.3.2). Methodological quality was assessed using the AMSTAR 2 tool, which comprises 16 core items, each rated as “Yes,” “Partial Yes,” “No,” or “No meta-analysis performed.” The evaluation was conducted online using the official AMSTAR Checklist (https://amstar.ca/Amstar_Checklist.php), and each SR was assigned an overall quality rating classified as “Critically Low,” “Low,” “Moderate,” or “High” (9). Reporting completeness was evaluated against the 27-item PRISMA statement, with each item categorized as “Fully Reported,” “Partially Reported,” or “Not Reported” (10). The GRADE system was employed to assess the certainty of evidence for the outcome measures. The final evidence quality grade was determined by systematically considering five key downgrading factors and three major upgrading factors, resulting in four final classifications: “High,” “Moderate,” “Low,” and “Critically Low” (11).

### 2.4 Evidence mapping

Currently, there are no standardized reporting guidelines or unified methodological criteria for evidence mapping. This study was therefore guided by the well-established Global Evidence Mapping methodology (12) and existing published evidence mapping research (13, 14). The evidence mapping results were visually presented via a four-dimensional bubble plot. In this plot, each bubble represents a SR, and the bubble size corresponds to the number of included primary studies. The X-axis represents the efficacy of TCM for HUA, while distinct bubble colors indicate different treatment methods. The Y-axis represents the methodological quality of the SRs. The bubble plot was generated using the online evidence mapping tool available on the Pymeta.com website (https://www.pymeta.com/evdmap/), which effectively illustrates the key characteristics of the synthesized studies.

### 2.5 Overlap analysis of primary studies

To address the potential for exaggerated evidence base due to the inclusion of identical primary RCTs across multiple SRs, we quantified the degree of overlap among included SRs using the Corrected Covered Area (CCA) index. A citation matrix encompassing all primary RCTs was constructed, and the CCA index was calculated using the following formula: *CCA = (N − r)/(r × c − r)*, where *N* denotes the total cumulative number of primary RCTs across all included SRs, *r* represents the count of unique non-duplicate primary RCTs, and *c* refers to the number of SRs in this evidence mapping. The CCA index indicates slight (0–5%), moderate (6–10%), high (11–15%), or very high (>15%) overlap.

## 3 Results

### 3.1 Literature retrieval and screening

**Figure 1:**
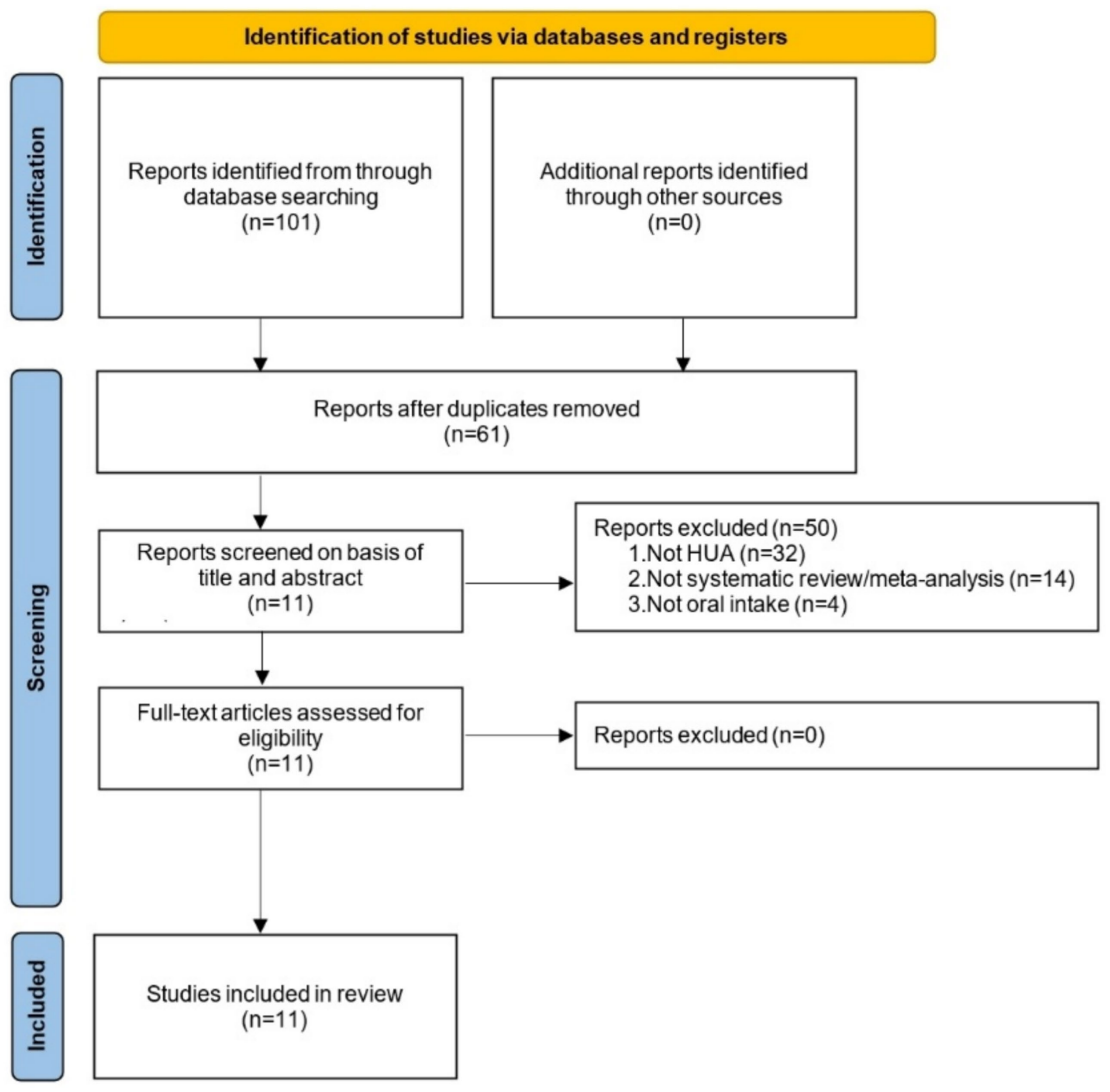
Literature screening process and results.

Initial database searches retrieved a total of 101 records. Specifically, 4 SRs were identified from PubMed, 0 from the Cochrane Library, 33 from Embase, 7 from WOS, 14 from CNKI, 8 from WanFang, 23 from SinoMed, and 12 from VIP. After duplicate records were removed, 61 unique records remained. Title and abstract screening was subsequently performed, leading to the exclusion of 50 records. Finally, a full-text assessment of the remaining eligible records was conducted, and 11 SRs were ultimately included in the present study. The detailed literature screening workflow is shown in Figure 1.

### 3.2 Characteristics of included studies

Table 1 summarizes the baseline characteristics of the 11 SRs, with regard to the study population, intervention regimens, and control interventions. These 11 SRs collectively encompassed 12,228 patients and were published between 2015 and 2025. Of these, 2 SRs were published in English, while the remaining 9 were published in Chinese. Interventions in the experimental groups consisted of either TCM monotherapy or ITCW, whereas the control groups received Western medicine (WM), placebo, or no treatment (blank control). Specifically, 6 studies included TCM monotherapy and 7 included integrated treatment. Furthermore, TCM therapeutic strategies were categorized as follows: single clearing dampness (Qushi, *n* = 5), clearing dampness and tonifying (Qushi-Buyi, *n* = 3), and clearing dampness combined with unblocking meridians (Qushi-Tongluo, *n* = 3).

**Table 1:** Characteristics of the included studies.

Study ID	Number of included RCTs (samples)	Treatment		Course of treatment	Outcome measures	Quality evaluation tools	Subgroup analysis	Sensitivity analysis	Funnel plot
		Experimental group	Control group						
Wang M, 2022 (17)	35 (2855)	TCM (Qushi)	WM	4–8 weeks	Total effective rate, sUA, incidence of adverse reactions	Cochrane	N	Y	N
Wu FS, 2017 (18)	10 (1127)	TCM (Qushi-Buyi)	WM, placebo	20 days–2 months	Short-term effective rate, long-term effective rate, short-term sUA, long-term sUA, incidence of adverse reactions	Cochrane	N	Y	N
Liu YJ, 2017 (19)	16 (1288)	ITCW (Qushi-Tongluo)	WM, placebo	3–48 weeks	Total effective rate, sUA, incidence of adverse reactions	Cochrane	N	Y	Y
Qiao LM, 2020 (16)	11 (731)	TCM (Qushi-Tongluo)	WM	22–32 weeks	sUA, incidence of adverse reactions	Jadad	N	N	N
Xu CX, 2025 (20)	13 (853)	ITCW (Qushi)	WM	14–90 days	Total effective rate, TCM syndrome score, sUA, Scr, incidence of adverse reactions	Cochrane	Y	N	N
Wu YY, 2024 (21)	8 (642)	TCM (*n* = 4), ITCW (*n* = 4) (Qushi-Buyi)	WM, placebo	4–12 weeks	Total effective rate, sUA, Scr, TG, TC	Cochrane	N	N	Y
Pan Y, 2024 (22)	11 (845)	ITCW (Qushi)	WM	8 weeks–4 months	Total effective rate, sUA, Scr, BUN, TNF-α	Cochrane	N	Y	N
Huo JJ, 2015 (23)	10 (1000)	ITCW (Qushi)	WM, placebo, blank	20 days–12 weeks	Total effective rate, Sua	Cochrane	N	N	Y
Shu ZM, 2017 (24)	14 (1374)	TCM (Qushi-Tongluo)	WM	>10 days	Total effective rate, incidence of adverse reactions	Jadad	N	Y	Y
Wang Y, 2022 (25)	6 (453)	ITCW (Qushi-Buyi)	WM	2–6 months	Sua	Cochrane	N	N	N
Chen LQ, 2020 (15)	10 (1060)	ITCW (*n* = 2), TCM (*n* = 8) (Qushi)	WM, placebo, blank	10 days–12 weeks	Sua	Cochrane	Y	Y	N

TCM, Traditional Chinese Medicine; ITCW, Integrated Traditional Chinese and Western medicine; WM, Western medicine; Qushi, single clearing dampness; Qushi-Buyi, clearing dampness and tonifying; Qushi-Tongluo, clearing dampness and unblocking meridians; sUA, serum uric acid; Scr, serum creatinine; TG, triglyceride; TC, total cholesterol; BUN, blood urea nitrogen; TNF-α, tumor necrosis factor-alpha; N, no; Y, yes.

Regarding methodological quality assessment, the Cochrane Risk of Bias (RoB) tool was applied in nine studies, while the Jadad scale was used in two studies. Additionally, subgroup analyses were conducted in two studies, six studies performed sensitivity analyses to test result robustness, and four studies generated funnel plots to evaluate potential publication bias. The comprehensive detailed characteristics of all included studies are fully presented in Table 1.

To quantify the extent of overlap among the 11 SRs, a citation matrix of the RCTs contained in these SRs was constructed (details are provided in Supplementary Table S2). A total of 144 primary studies were cited across all SRs, and 102 unique primary RCTs were identified after deduplication. The CCA index was calculated as 4.12% using the standard formula, indicating a slight overlap within the evidence base. This low overlap confirms that only a small proportion of primary studies were repeatedly cited, and the 11 SRs predominantly synthesized distinct and independent sets of original clinical evidence. This evidence distribution pattern may be attributed to the heterogeneity in TCM therapeutic strategies evaluated (i.e., Qushi, Qushi-Buyi, and Qushi-Tongluo therapies) and the broad publication time span of the included SRs (2015–2025). Accordingly, the pooled sample size of 12,228 patients reasonably reflects the comprehensive scope of the available clinical evidence base, suggesting that the findings of this study were not artificially inflated by redundant data extraction across the included SRs.

### 3.3 Quality assessment of included systematic reviews and meta-analyses

#### 3.3.1 Results of methodological quality assessment

**Figure 2:**
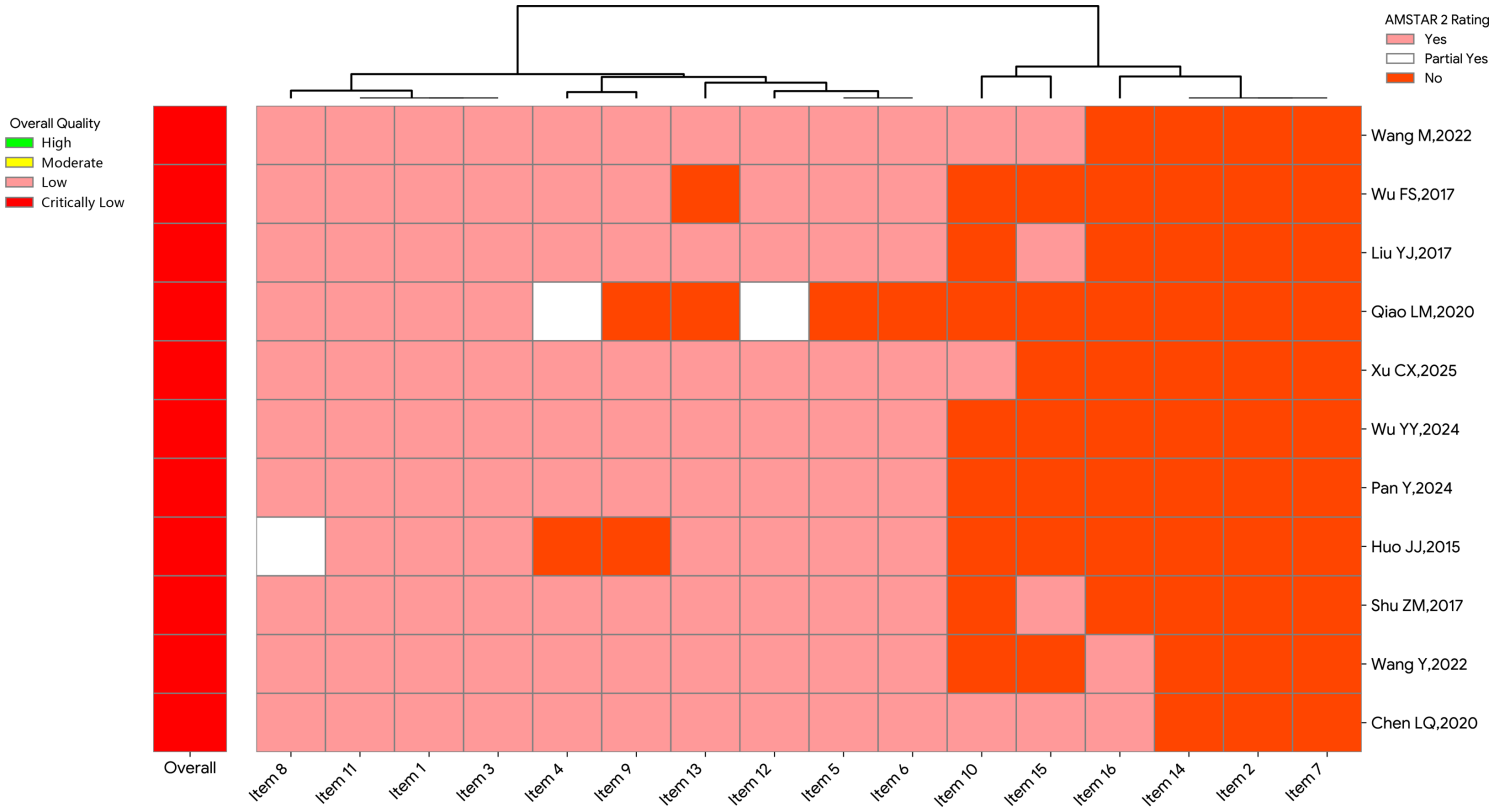
Heatmap of the methodological quality assessment of SRs.

The methodological quality of the 11 included SRs was rigorously appraised using the AMSTAR 2 tool, with detailed findings presented in Table 2 and Figure 2. The overall quality assessment revealed that all 11 studies (100%) were graded with an overall confidence rating of “Critically Low.” Regarding reporting completeness, Items 1, 3, 4, 5, 6, 8, 9, 11, 12, and 13 demonstrated satisfactory reporting quality, with completeness rates exceeding 70%. Specifically, all 11 studies (100%) clearly defined the research question using the PICO framework and adequately described the study design and statistical synthesis methods (Items 1, 3, and 11, 100%). Furthermore, the majority of studies exhibited acceptable performance in terms of comprehensive literature search strategy (Item 4, 82%), screening and data extraction processes (Items 5 and 6, 91%), description of baseline characteristics for included primary studies (Item 8, 91%), and risk of bias assessment (Item 9, 82%). Nevertheless, critical limitations were identified in core methodological domains: None of the SRs provided pre-registered protocol information (Item 2, 0%) or a list of excluded studies with justifications (Item 7, 0%). These omissions severely undermine the methodological transparency and replicability of the research process. Non-critical items also showed universal deficits, as all SRs failed to adequately explain the sources of heterogeneity (Item 14, 0%), and with the exception of Chen et al. (15), none of the remaining SRs adequately disclosed conflicts of interest and funding sources (Item 16, 9.1%).

**Table 2:** Results of methodological quality assessment of SRs (AMSTAR 2).

Study ID	Item 1	Item 2*	Item 3	Item 4*	Item 5	Item 6	Item 7*	Item 8	Item 9*	Item 10	Item 11*	Item 12	Item 13*	Item 14	Item 15*	Item 16	Overall
Wang M, 2022 (17)	Y	N	Y	Y	Y	Y	N	Y	Y	Y	Y	Y	Y	N	Y	N	Critically low
Wu FS, 2017 (18)	Y	N	Y	Y	Y	Y	N	Y	Y	Y	Y	Y	Y	N	Y	N	Critically low
Liu YJ, 2017 (19)	Y	N	Y	Y	Y	Y	N	Y	Y	N	Y	Y	N	N	N	N	Critically low
Qiao LM, 2020 (16)	Y	N	Y	Y	Y	Y	N	Y	Y	N	Y	Y	Y	N	Y	N	Critically low
Xu CX, 2025 (20)	Y	N	Y	PY	N	N	N	Y	N	N	Y	PY	N	N	N	N	Critically low
Wu YY, 2024 (21)	Y	N	Y	Y	Y	Y	N	Y	Y	Y	Y	Y	Y	N	N	N	Critically low
Pan Y, 2024 (22)	Y	N	Y	Y	Y	Y	N	Y	Y	N	Y	Y	Y	N	N	N	Critically low
Huo JJ, 2015 (23)	Y	N	Y	Y	Y	Y	N	Y	Y	N	Y	Y	Y	N	N	N	Critically low
Shu ZM, 2017 (24)	Y	N	Y	N	Y	Y	N	PY	N	N	Y	Y	Y	N	N	N	Critically low
Wang Y, 2022 (25)	Y	N	Y	Y	Y	Y	N	Y	Y	N	Y	Y	Y	N	Y	N	Critically low
Chen LQ, 2020 (15)	Y	N	Y	Y	Y	Y	N	Y	Y	N	Y	Y	Y	N	N	Y	Critically low

The seven critical items are designated as 2, 4, 7, 9, 11, 13, and 15. The assessment categories are defined as follows: Y (Yes), PY (Partially Yes), and N (No). A rating of “Critically Low” is assigned when more than one critical item is non-compliant, regardless of the compliance status of non-critical items.

#### 3.3.2 Results of reporting quality assessment

**Figure 3:**
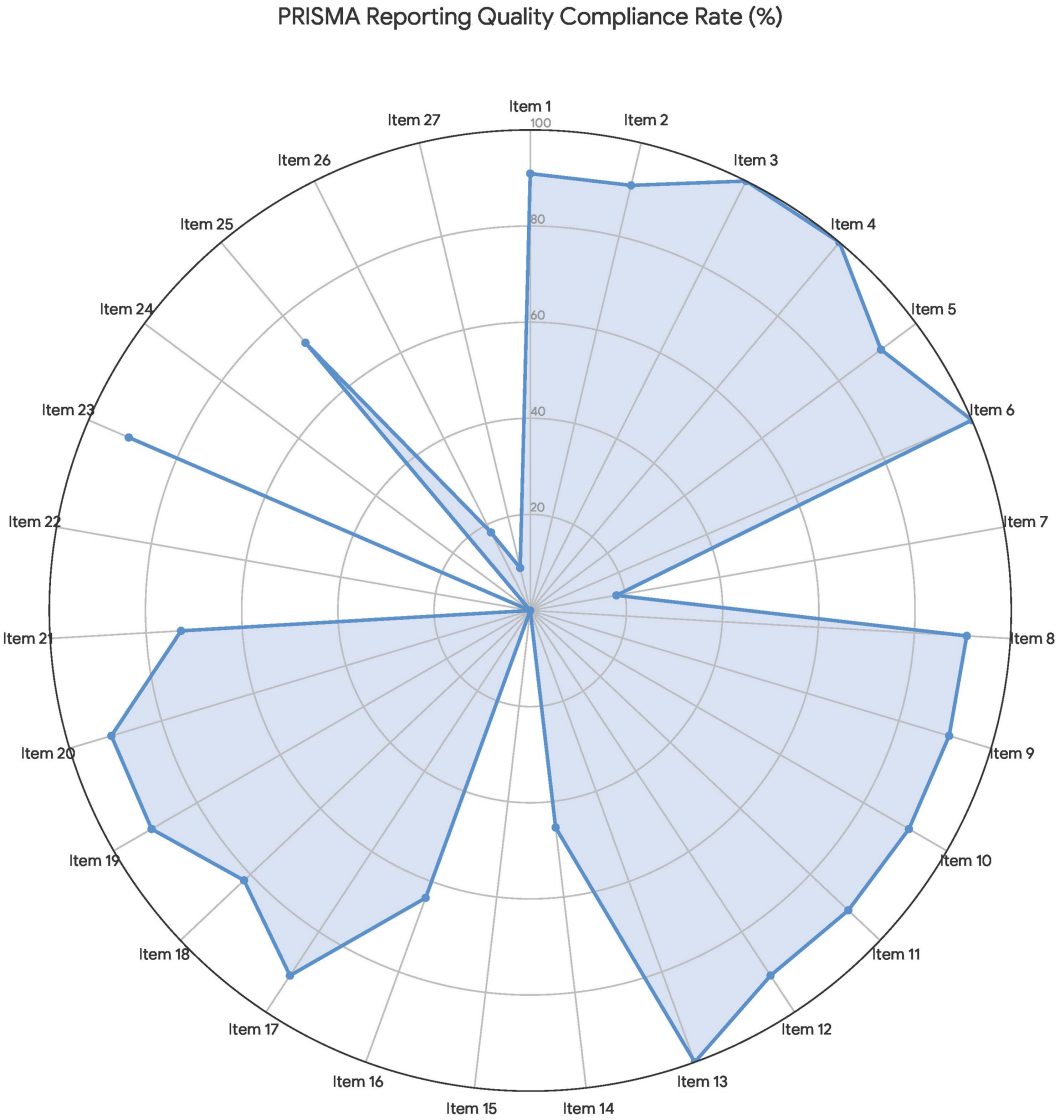
Radar chart of the evaluation results of PRISMA reporting quality.

**Figure 4:**
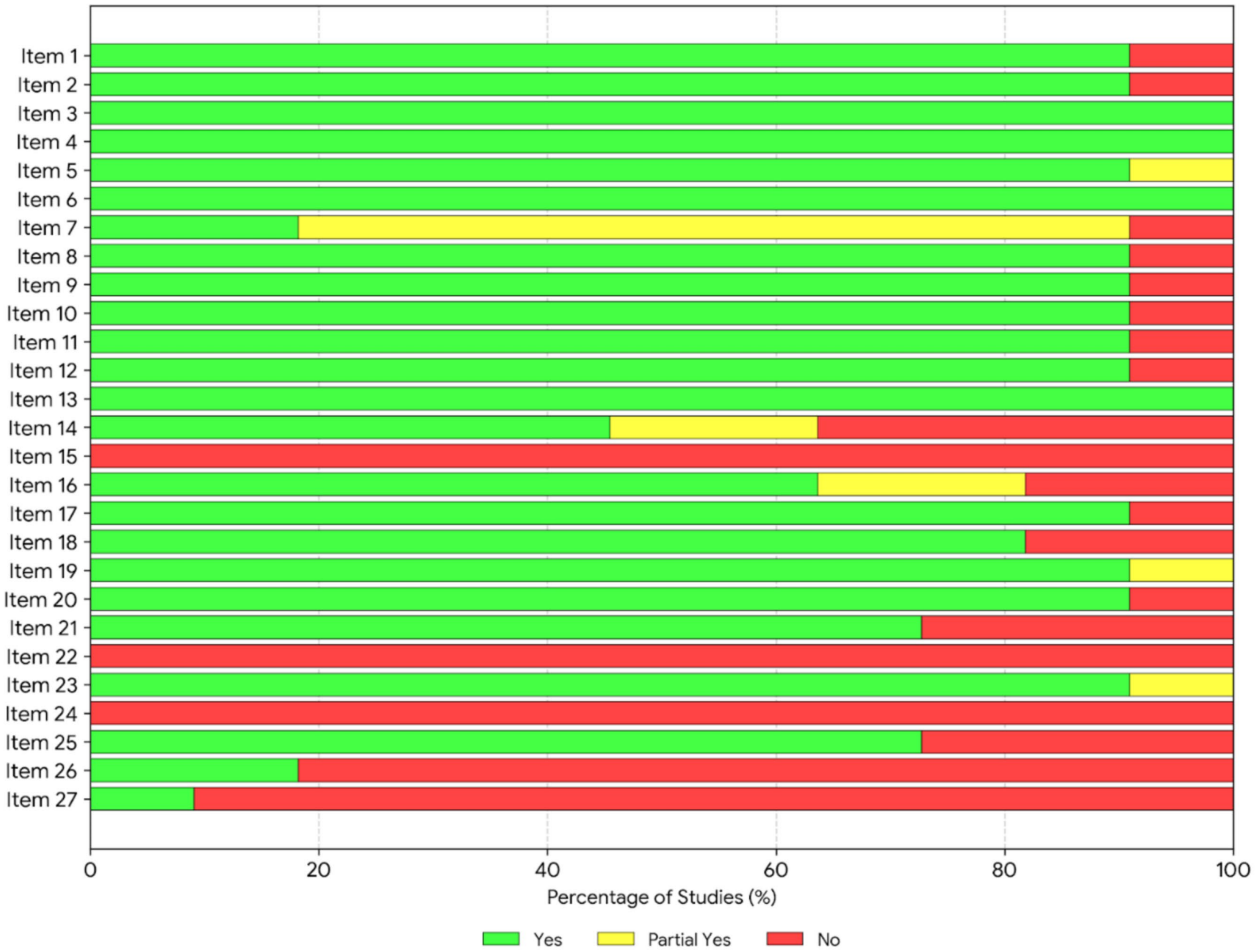
Stacked bar chart of PRISMA reporting quality.

The reporting quality of the 11 included SRs was evaluated in accordance with the PRISMA statement, with detailed findings documented in Table 3 and Figures 3, 4. The included SRs exhibited substantial heterogeneity in overall compliance with the PRISMA checklist items, with the maximum compliance rate reaching 88.89% and the minimum rate reaching as low as 29.63%. While the PRISMA statement does not recommend a categorical rating scale for reporting quality, an item-level analysis identified a pronounced polarization in reporting quality across the included studies. Detailed item-by-item assessment demonstrated that, although all included studies achieved full reporting completeness for descriptive core information, including theoretical rationale and research objectives (Items 1–6; 100% reporting rate), systemic deficiencies remained prevalent in core domains associated with research transparency and methodological rigor. Specifically, seven critical PRISMA items had a reporting completeness rate below 50%, indicating substantial information gaps according to the PRISMA reporting guidelines. Crucially, Item 15 (Certainty of evidence assessment), Item 22 (Overall certainty of evidence synthesis), and Item 24 (Study registration and protocol access) were not reported in any of the included SRs (0% reporting rate), indicating that none of the SRs provided a preregistration protocol number or conducted the required robustness and validity verification of the synthesized evidence. Additionally, several key items were consistently underreported across the included studies: Item 7 (Systematic literature search strategy, 18.2%), Item 14 (Publication and reporting bias assessment, 45.5%), Item 26 (Competing interests disclosure, 18.2%), and Item 27 (Availability of raw data, analytical code, and supplementary research materials, 9.1%). This widespread underreporting trend was further corroborated by the distribution of “Not Reported (red)” and “Partially Reported (yellow)” classifications illustrated in Figure 3. Individual study scrutiny confirmed that only the study by Chen et al. (15) fully complied with the PRISMA reporting standards for funding sources and competing interests disclosure. In contrast, the study by Qiao and Li (16), which achieved the lowest overall compliance score, contained extensive methodological omissions covering literature search, study screening, and bias assessment, representing the lowest level of methodological reproducibility and transparency among all included SRs.

**Table 3:** Assessment of reporting quality for SRs (PRISMA).

Study ID	Item 1	Item 2	Item 3	Item 4	Item 5	Item 6	Item 7	Item 8	Item 9	Item 10	Item 11	Item 12	Item 13	Item 14	Item 15	Item 16	Item 17	Item 18	Item 19	Item 20	Item 21	Item 22	Item 23	Item 24	Item 25	Item 26	Item 27	Compliance rate
Wang M, 2022 (17)	Y	Y	Y	Y	Y	Y	PY	Y	Y	Y	Y	Y	Y	Y	N	Y	Y	Y	Y	Y	Y	N	Y	N	Y	N	N	77.78%
Wu FS, 2017 (18)	Y	Y	Y	Y	Y	Y	PY	Y	Y	Y	Y	Y	Y	PY	N	Y	Y	Y	Y	Y	Y	N	Y	N	Y	N	N	74.07%
Liu YJ, 2017 (19)	Y	Y	Y	Y	Y	Y	PY	Y	Y	Y	Y	Y	Y	Y	N	PY	Y	Y	Y	Y	Y	N	Y	N	Y	N	N	74.07%
Qiao LM, 2020 (16)	Y	Y	Y	Y	PY	Y	N	N	N	Y	N	N	Y	N	N	PY	Y	N	PY	N	N	N	PY	N	N	N	N	29.63%
Xu CX, 2025 (20)	Y	Y	Y	Y	Y	Y	PY	Y	Y	Y	Y	Y	Y	N	N	Y	Y	Y	Y	Y	N	N	Y	N	Y	N	N	70.37%
Wu YY, 2024 (21)	Y	Y	Y	Y	Y	Y	PY	Y	Y	Y	Y	Y	Y	N	N	Y	Y	Y	Y	Y	Y	N	Y	N	Y	N	N	74.07%
Pan Y, 2024 (22)	Y	Y	Y	Y	Y	Y	PY	Y	Y	Y	Y	Y	Y	PY	N	Y	Y	Y	Y	Y	Y	N	Y	N	Y	N	N	74.07%
Huo JJ, 2015 (23)	Y	Y	Y	Y	Y	Y	Y	Y	Y	Y	Y	Y	Y	Y	N	Y	Y	Y	Y	Y	Y	N	Y	N	N	N	N	77.78%
Shu ZM, 2017 (24)	Y	N	Y	Y	Y	Y	PY	Y	Y	Y	Y	Y	Y	Y	N	N	Y	N	Y	Y	Y	N	Y	N	N	N	N	62.96%
Wang Y, 2022 (25)	N	Y	Y	Y	Y	Y	PY	Y	Y	N	Y	Y	Y	N	N	N	N	Y	Y	Y	N	N	Y	N	Y	Y	N	59.26%
Chen LQ, 2020 (15)	Y	Y	Y	Y	Y	Y	Y	Y	Y	Y	Y	Y	Y	Y	N	Y	Y	Y	Y	Y	Y	N	Y	N	Y	Y	Y	88.89%

Compliance rate is the number of compliant entries divided by the total number of entries.

#### 3.3.3 Statistical analysis of outcome measures

A total of eight included SRs (17–24) evaluated the total effective rate and generated corresponding forest plots for quantitative synthesis. Of these studies, five SRs (17, 18, 21, 23, 24) verified that the TCM group yielded a significantly higher total effective rate than the WM group, while four SRs (19–22) demonstrated that the ITCW group exhibited superior efficacy than the WM or placebo group. Notably, one SR (18) conducted separate subgroup analyses for short-term and long-term efficacy rates and concluded that the TCM group achieved consistently higher efficacy rates than the WM group across both follow-up periods.

Furthermore, 10 SRs (15–23, 25) performed pooled analyses of sUA levels. Among these SRs, six (15–18, 21, 23) confirmed that the TCM group exerted a more significant therapeutic effect on reducing sUA levels relative to the WM, placebo, or blank control group, and six SRs (15, 19–22, 25) documented that the ITCW group achieved superior sUA-lowering efficacy than the WM or placebo group. Additionally, Wu et al. (18) specifically investigated short-term and long-term sUA reduction effects and revealed that the TCM group resulted in a greater magnitude of sUA reduction than the WM group at both time points.

Moreover, one SR (20) conducted a meta-analysis of TCM syndrome scores, and the findings indicated that TCM administration significantly ameliorated hyperuricemia-related TCM syndrome scores, corresponding to a remarkable reduction in clinical syndrome severity.

Regarding safety outcomes, three SRs (15, 19, 24) performed a descriptive analysis of adverse drug reactions, with the main reported adverse events including lumbar pain, epigastric discomfort, and abnormal hepatic and renal function. A total of six SRs (16–20, 24) quantitatively analyzed the incidence of adverse reactions. Among these six, three SRs (16, 17, 24) documented a significantly lower incidence of adverse reactions in the TCM group relative to the WM group, whereas one SR (18) reported no statistically significant between-group difference in adverse reaction incidence. Additionally, two SRs (19, 20) demonstrated that the ITCW group had a lower adverse event rate compared with the WM group, and one SR (19) found no significant difference in the incidence of adverse event between the ITCW group and the placebo group.

#### 3.3.4 Results of GRADE certainty assessment

The certainty of evidence for the multiple outcome measures extracted from the 11 included SRs was appraised using the GRADE system, with detailed assessment findings comprehensively presented in Table 4. Overall, the certainty of evidence in this clinical research field was uniformly low, indicating that the true underlying therapeutic effect of current oral TCM interventions for HUA may differ substantially from the pooled effect estimates, thereby resulting in low confidence in the synthesized research findings. Among all pooled outcome measures, only the outcome of “adverse event incidence” reported by Wang et al. (17) was graded as moderate certainty evidence, while all remaining outcome measures were rated as either “low” or “critically low” certainty. An in-depth analysis identified that study “Limitations” (risk of bias) constituted the primary factor contributing to evidence downgrading, which impacted all assessed outcome measures. This widespread downgrading was primarily attributed to pervasive methodological deficiencies in the original RCTs, particularly inadequate reporting of random sequence generation, allocation concealment, and blinding implementations. Second, “Publication Bias” exerted a substantial impact on evidence certainty: The majority of the outcome measures were downgraded by one level owing to asymmetric funnel plot distributions or unexcludable potential publication bias, with the exception of several specific outcomes reported in the studies by Chen et al. (15) and Wu et al. (18). Furthermore, during the certainty assessment of objective biochemical indicators (including sUA reduction and serum creatinine (Scr) levels), “Inconsistency” and “Imprecision” were frequent contributors to evidence downgrading, reflecting marked between-study heterogeneity in effect magnitudes and insufficient total pooled sample sizes across the included primary studies. Notably, no outcome measures were downgraded due to “Indirectness,” which verifies that the PICO components of the existing evidence base are highly consistent with real-world clinical diagnosis and treatment scenarios, thereby offering clinically relevant evidence support for both scientific research and routine clinical practice.

**Table 4:** Results of the GRADE assessment of evidence quality.

Study ID	Outcome measures	RCTs	Limitations	Inconsistency	Indirectness	Imprecision	Publication bias	Level
Wang M, 2022 (17)	Total effective rate, TCM VS WM	35	↓	-	-	-	↓	Low
	sUA, TCM VS WM	35	↓	↓	-	-	↓	Critically low
	Incidence of adverse reactions, TCM VS WM	25	↓	-	-	-	-	Moderate
Wu FS, 2017 (18)	Short-term total effective rate, TCM VS WM	8	↓↓	-	-	-	↓	Critically low
	Short-term total effective rate, TCM VS placebo	1	↓↓	-	-	-	↓	Critically low
	Long-term total effective rate, TCM VS WM	3	↓↓	-	-	-	↓	Critically low
	Short-term sUA, TCM VS WM	7	↓↓	↓	-	↓	↓	Critically low
	Long-term sUA, TCM VS WM	3	↓↓	↓↓	-	-	↓	Critically low
	Incidence of adverse reactions, TCM VS WM	4	↓↓	-	-	-	-	Low
Liu YJ, 2017 (19)	Total effective rate, ITCW VS WM	10	↓	-	-	-	↓	Low
	Total effective rate, ITCW VS placebo	4	↓	↓	-	-	↓	Critically low
	sUA, ITCW VS WM	11	↓	↓↓	-	-	↓	Critically low
	sUA, ITCW VS placebo	5	↓	↓	-	-	↓	Critically low
	Incidence of adverse reactions, ITCW VS WM	7	↓↓	-	-	-	↓	Critically low
	Incidence of adverse reactions, ITCW VS placebo	3	↓	-	-	↓	↓	Critically low
Qiao LM, 2020 (16)	sUA, TCM VS WM	11	↓↓	↓	-	↓	↓	Critically low
	Incidence of adverse reactions, TCM VS WM	11	↓↓	-	-	-	↓	Critically low
Xu CX, 2025 (20)	Total effective rate, ITCW VS WM	9	↓↓	-	-	-	↓	Critically low
	TCM syndrome score, ITCW VS WM	3	↓↓	-	-	↓	↓	Critically low
	sUA, ITCW VS WM	8	↓↓	↓	-	-	↓	Critically low
	Scr, ITCW VS WM	4	↓↓	-	-	↓	↓	Critically Low
	Incidence of adverse reactions, ITCW VS WM	5	↓↓	-	-	↓↓	↓	Critically low
Wu YY, 2024 (21)	Total effective rate, TCM VS WM	4	↓	↓	-	↓	↓	Critically low
	Total effective rate, ITCW VS WM	4	↓	-	-	↓	↓	Critically low
	sUA, TCM VS WM/placebo	4	↓	-	-	↓	↓	Critically low
	sUA, ITCW VS WM	4	↓	↓	-	↓	↓	Critically low
	Scr, TCM/ITCW VS placebo/WM	4	↓	-	-	↓	↓	Critically low
	TG, TCM/ITCW VS placebo/WM	4	↓	↓	-	↓	↓	Critically low
	TC, TCM/ITCW VS placebo/WM	3	↓	↓	-	↓	↓	Critically low
Pan Y, 2024 (22)	Total effective rate, ITCW VS WM	11	↓	-	-	-	↓	Low
	sUA, ITCW VS WM	11	↓	↓↓	-	↓	↓	Critically low
	Scr, ITCW VS WM	6	↓	↓↓	-	↓	↓	Critically low
	BUN, ITCW VS WM	5	↓	↓↓	-	↓	↓	Critically low
	TNF-α, ITCW VS WM	3	↓	↓	-	↓↓	↓	Critically low
Huo JJ, 2015 (23)	Total effective rate, TCM VS WM	10	↓↓	-	-	-	↓	Critically low
	sUA, TCM VS WM	10	↓↓	↓	-	-	↓	Critically low
Shu ZM, 2017 (24)	Total effective rate, TCM VS WM	14	↓↓	↓	-	-	↓	Critically low
	Incidence of adverse reactions, TCM VS WM	10	↓	-	-	↓	↓	Critically low
Wang Y, 2022 (25)	sUA, ITCW VS WM	6	↓	-	-	-	↓	Low
Chen LQ, 2020 (15)	sUA, TCM VS placebo/blank	4	↓	↓↓	-	↓	-	Critically low
	sUA, TCM VS WM	6	↓	↓↓	-	↓	-	Critically low
	sUA, ITCW VS WM	2	↓	↓↓	-	↓↓	-	Critically low

↓, degraded by 1 level. ↓↓, degraded by 2 level. -, no degraded.

### 3.4 Results of evidence mapping

**Figure 5:**
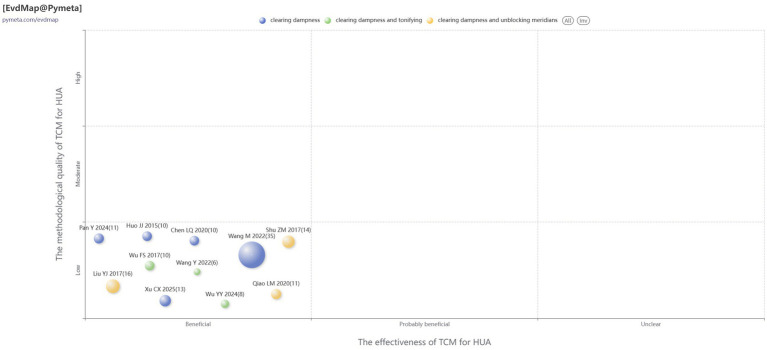
Bubble plot of the evidence mapping of included SRs.

Figure 5 presented a bubble plot characterizing the methodological quality and corresponding evidence certainty of the 11 SRs concerning TCM for HUA. Efficacy evaluation demonstrated that all included SRs were distributed within the “effective” efficacy range, which consistently indicates that available evidence supports the favorable therapeutic effects of TCM in lowering sUA levels and ameliorating clinical symptoms associated with HUA. Nevertheless, an analysis of methodological quality revealed that the overall standard of current research remained low, indicating significant room for improvement in aspects such as study design and bias control.

The oral TCM therapeutic regimens for HUA management were stratified into three distinct categories: five SRs exclusively focused on Qushi (single clearing dampness) therapy (15, 17, 20, 22, 23), three targeted Qushi-Buyi (clearing dampness and tonifying) therapy (18, 21, 25), and three investigated Qushi-Tongluo (clearing dampness and unblocking meridians) therapy (16, 19, 24). Collectively, Qushi constitutes the fundamental therapeutic principle underlying all included TCM interventions. Variations in bubble size correspond to the number of included primary studies, with larger bubbles indicating more primary RCTs synthesized: Studies implementing single Qushi therapy corresponded to a markedly larger cumulative sample size (blue bubbles: 79 RCTs, 6,613 participants). In contrast, SRs evaluating combined therapeutic principles, namely Qushi-Buyi (green bubbles: 24 RCTs, 2,222 participants) and Qushi-Tongluo (yellow bubbles: 41 RCTs, 3,393 participants), encompassed fewer primary studies and smaller pooled sample sizes.

Importantly, a larger bubble size merely reflects a greater number of included primary studies and does not indicate high-certainty evidence; this correlation is further reinforced by the fact that the methodological quality of all included SRs was uniformly graded as critically low. Although these combined TCM therapeutic strategies exhibited promising clinical benefits, the limited pooled sample sizes necessitate further rigorous validation to confirm the stability and reliability of their pooled effect estimates. In conclusion, the currently available evidence supports the core role of Qushi-based therapeutic principles in the clinical management of HUA. Nevertheless, pervasive methodological flaws across included SRs and the relative paucity of high-quality combination therapy research highlight the urgency for future prospective studies with larger sample cohorts and more rigorous methodological designs. Such studies are warranted to precisely delineate the differences in efficacy among diverse Qushi-based combination strategies, thereby establishing a more robust and reliable evidence foundation to guide the clinical application of TCM interventions for HUA treatment.

## 4 Discussion

### 4.1 Therapeutic advantages and clinical safety of TCM for HUA

Our study utilized evidence mapping to visualize the existing evidence base for TCM interventions for HUA. The results demonstrate consistency among the conclusions of the included SRs, despite variations in publication year and sample size. Specifically, TCM was superior to WM, placebo, or no treatment, and ITCW was superior to conventional Western medicine or placebo, in terms of reducing sUA levels and improving overall clinical response rates.

Such consistency suggests that TCM’s mechanism—intervening in uric acid metabolism through multi-target and multi-pathway regulation—holds reproducible clinical value, which is distinct from the single-target intervention commonly used in Western medicine. In theory, ITCW may achieve complementary and synergistic effects compared with Western medicine alone. However, given the critically low methodological quality of the synthesized evidence, these findings warrant cautious clinical interpretation, and TCM treatments should strictly adhere to pattern differentiation to ensure clinical rationality.

In line with classical TCM theory, the pathogenesis of HUA is predominantly ascribed to the “accumulation of dampness-turbidity and obstruction by phlegm-stasis”. Since dampness-turbidity is viscous and difficult to resolve, its chronic presence may obstruct meridians and impair visceral function. Therefore, core clinical strategies focus on clearing dampness to unblock meridians (Qushi-Tongluo) or clearing dampness combined with tonification (Qushi-Buyi). This multi-target approach—which encompasses promoting diuresis, expelling turbidity, activating blood circulation, and tonifying the Spleen and Kidney—is proposed to modulate both uric acid synthesis and excretion. Such mechanisms align closely with the complex pathogenesis of HUA, characterized by interwoven dampness and stasis alongside mixed deficiency and excess. The evidence mapping highlights “clearing dampness” as a critical therapeutic method, which corresponds both to the core pathogenesis and the holistic regulatory strategy of treating tip while consolidating the root. Furthermore, modern pharmacological research indicates that formulas based on the dampness-eliminating principle can inhibit uric acid production by modulating xanthine oxidase activity, promote excretion by upregulating uric acid transporters (e.g., URAT1 and GLUT9), and simultaneously reduce renal inflammation and oxidative stress damage. These actions provide objective biological evidence for the observed clinical efficacy by blocking the molecular pathways related to dampness-turbidity (26).

Crucially, the safety profile of TCM interventions was found to be comparable to or better than that of the WM group, with adverse events being predominantly mild gastrointestinal reactions and no evidence of severe hepatorenal toxicity (27–29). Nevertheless, because adverse event data were not systematically collected or completely reported across most primary RCTs, and liver/kidney toxicity from the incorrect administration of TCM is sometimes reported clinically, it is more accurate to state that the available data do not suggest major safety concerns, rather than claiming a definitive favorable safety profile. However, this potential safety advantage remains highly significant, given that the long-term use of conventional drugs, such as allopurinol or benzbromarone, is often limited by associated adverse event risks. Given its characteristics of simplicity, cost-effectiveness, and superior safety, TCM may become an essential component in the long-term, integrated management of HUA, particularly for patients intolerant to Western drugs or those in the asymptomatic stage.

### 4.2 Deficiencies in methodological quality and reporting standards of the existing evidence base

Despite the encouraging clinical efficacy findings, our rigorous assessment utilizing the AMSTAR 2, PRISMA, and GRADE tools revealed significant limitations in the quality of evidence within this field. These limitations represent the primary barrier preventing the adoption of TCM evidence by high-level international guidelines. First, severe methodological deficiencies were identified. The 11 included SRs were uniformly rated as critically low by AMSTAR 2. The most critical issues involved the absence of pre-registered protocols and the failure to provide a list of excluded studies. This lack of pre-registration severely compromises the transparency of core stages, including study design and statistical analysis, potentially leading to selective reporting bias driven by outcome-oriented analytical adjustments (30). Second, reporting quality exhibited structural gaps. While PRISMA scores indicated satisfactory reporting for descriptive items, crucial areas concerning research rigor—such as cross-study bias assessment, funding sources, and conflicts of interest declarations—were largely incomplete. GRADE profiling demonstrated that the certainty of evidence for the majority of outcomes was low or critically low. The main contributors to this downgrading of certainty were unclear allocation concealment in the original RCTs and substantial inter-study heterogeneity. The positive findings supported by this low-quality evidence necessitate a cautious interpretation of efficacy as we cannot rule out that the perceived efficacy advantage is partly attributable to methodological biases inherent in the low-quality primary studies.

### 4.3 Implications for future evidence-based research and clinical decision-making

Based on these findings, future research efforts should shift from prioritizing study quantity to emphasizing methodological quality. For RCT investigators, rigorous adherence to the CONSORT Statement is essential (31). Efforts should focus particularly on enhancing the execution and reporting of randomization methods, allocation concealment, and blinding procedures, thereby improving the inherent validity of the evidence. For systematic reviewers and meta-analysts, prospective registration on platforms such as PROSPERO is mandatory, alongside strict compliance with the PRISMA 2020 guidelines during manuscript preparation. Special attention should be paid to exploring sources of heterogeneity and comprehensively evaluating publication bias to prevent the generation of redundant, low-quality data (32). Our evidence mapping indicates that current research predominantly assesses short-term efficacy, showing insufficient focus on long-term prognostic follow-up and health economic evaluation. We recommend that subsequent studies investigate the long-term benefits of TCM for HUA (e.g., frequency of gout flares and cardiovascular event risk) and conduct robust cost-effectiveness analyses. At the clinical decision-making level, the present study indicates that TCM may exert a uric acid-lowering effect and exhibits favorable safety potential. Nevertheless, given the generally low quality of evidence derived from the included studies, the findings should be interpreted with caution. Clinicians may consider TCM as a foundational or adjunctive therapeutic strategy for HUA after comprehensive patient evaluation. Meanwhile, practitioners are advised to fully inform patients of the limitations inherent in the current evidence base, perform regular monitoring of relevant laboratory indicators, and carry out flexible individualized adjustments in line with the core principles of TCM, such as pattern differentiation and holistic multi-dimensional intervention, thereby facilitating personalized and precise clinical management.

## 5 Conclusion

We employed an umbrella systematic review combined with evidence mapping to systematically summarize the SRs on TCM for HUA. Our findings indicate that TCM interventions primarily adopt the clearing dampness therapeutic principle, demonstrating potential benefits in lowering serum uric acid levels, alleviating clinical symptoms, and exhibiting a safety profile. Nevertheless, the existing evidence base currently is characterized by generally low certainty, constrained by methodological limitations in the primary studies and the systematic reviews themselves (e.g., deficiencies in protocol registration, risk of bias control, and adherence to reporting standards). Although the current results support TCM’s clinical utility, they are insufficient to inform strong clinical recommendations. Moving forward, high-quality clinical trials featuring rigorous design, proper registration, and transparent reporting are urgently required to strengthen the evidence-based foundation for TCM in the management of metabolic diseases.

## 6 Strengths and limitations

This study is the first to employ evidence mapping combined with an umbrella review to visualize the landscape of TCM for hyperuricemia, while rigorously assessing methodological quality and evidence certainty using AMSTAR 2, PRISMA, and GRADE tools. However, several limitations must be acknowledged. First, the “Critically Low” methodological quality of the included SRs, the pervasive risk of bias in primary RCTs, and the overlap of RCTs included in SRs limited the overall credibility of the study results. Second, the external validity and clinical extrapolation potential of our study conclusions are limited by two core factors. To mitigate confounding bias associated with baseline metabolic status, we excluded SRs focusing on patients with comorbid chronic underlying diseases such as diabetes mellitus, which restricts the direct extrapolation of our findings to real-world clinical settings where HUA frequently co-occurs with complex metabolic syndrome. Additionally, the vast majority of the included studies were conducted in China, resulting in significant geographic concentration of the study data, which further constrains the general applicability of our conclusions. Finally, the inherent heterogeneity of TCM formulations presents challenges for precise cross-intervention comparisons.

## Data Availability

The original contributions presented in the study are included in the article/Supplementary material, further inquiries can be directed to the corresponding author.
