# Supplementary material for: An evidence mapping study based on systematic reviews of traditional Chinese medicine for hyperuricemia
Source: Front Nutr. 2026 Apr 23;13:1811252. doi: 10.3389/fnut.2026.1811252 (PMC13149202; doi:10.3389/fnut.2026.1811252)
Supplement: Supplementary file 1 [file Table_1.DOCX]

Supplementary Material

# Supplementary Figures and Tables

## Supplementary Table

**Supplementary Table S1. Search strategies for all databases.**

1. PubMed (n=4)

| #1 | "Hyperuricemia"[Mesh] |
| --- | --- |
| #2 | hyperuricemia [Title/Abstract] |
| #3 | #1 OR #2 |
| #4 | "Medicine, Chinese Traditional"[Mesh] |
| #5 | (Traditional Chinese Medicine[Title/Abstract]) OR (Zhong Yi Xue[Title/Abstract]) OR (Chinese Traditional Medicine[Title/Abstract]) OR (traditional Chinese drug[Title/Abstract]) OR (Chinese herbal medicine[Title/Abstract]) OR (Chinese medicine[Title/Abstract]) OR (Chinese patent medicine[Title/Abstract]) |
| #6 | #4 OR #5 |
| #7 | "Meta-Analysis" [Publication Type] |
| #8 | (((systematic review[Title/Abstract]) OR (Metaanalysis[Title/Abstract])) OR (Meta-analysis[Publication Type])) OR(systematic review[Publication Type]) |
| #9 | "Systematic Reviews as Topic"[Mesh] |
| #10 | #7 OR #8 OR #9 |
| #11 | #3 AND #6 AND #10 |

1. The Cochrane Library: (n=0)

| #1 | MeSH descriptor: [Medicine, Chinese Traditional] explode all trees |
| --- | --- |
| #2 | MeSH descriptor: [Hyperuricemia] explode all trees |
| #3 | #1 AND #2 |

1. Embase: (n=33)

| #1 | 'meta analysis'/exp |
| --- | --- |
| #2 | 'meta analysis (topic)'/exp |
| #3 | 'systematic review'/exp |
| #4 | 'systematic review (topic)'/exp |
| #5 | 'systematic review':ab,ti OR 'meta analysis':ab,ti OR 'meta analyses':ab,ti OR 'meta-analysis':ab,ti OR 'meta-analyses':ab,ti OR metaanalysis:ab,ti OR metanalysis:ab,ti OR metaanalyse:ab,ti OR metanalyses:ab,ti |
| #6 | #1 OR #2 OR #3 OR #4 OR #5 |
| #7 | 'Traditional Chinese Medicine':ab,ti OR 'Chinese Traditional Medicine':ab,ti OR 'traditional Chinese drug':ab,ti OR 'Chinese herbal medicine':ab,ti OR 'Chinese medicine':ab,ti OR 'Chinese patent medicine':ab,ti |
| #8 | #6 AND #7 |
| #9 | 'hyperuricemia'/exp |
| #10 | 'hyperuricemia (topic)'/exp |
| #12 | #9 OR #10 |
| #11 | #8 AND #12 |

1. WOS: (n=7)

| #1 | TS=("systematic review" OR “meta-analysis” or metaanalysis OR “meta analysis” OR “meta analyses” OR “meta-analysis” OR “meta-analyses” OR metaanalysis OR metanalysis OR metaanalyse OR metanalyses) AND TS=("Traditional Chinese Medicine" or"traditional Chinese drug" or"Chinese herbal medicine" or "Chinese medicine" or"Chinese patent medicine") AND TS=(" hyperuricemia") |
| --- | --- |

1. SinoMed: (n=23)

| #1 | ("系统评价"[常用字段:智能] OR "meta分析"[常用字段:智能] OR "荟萃分析"[常用字段:智能] OR "系统综述"[常用字段:智能]) |
| --- | --- |
| #2 | "Meta分析"[不加权:扩展] |
| #3 | (#2) OR (#1) |
| #4 | ("TCM "[常用字段:智能] OR "中医"[常用字段:智能] OR "中医药"[常用字段:智能] OR "草药"[常用字段:智能] OR "中成药"[常用字段:智能] OR "制剂"[常用字段:智能] OR "方剂"[常用字段:智能] OR "复方"[常用字段:智能] ) |
| #5 | "高尿酸血症"[常用字段:智能] |
| #6 | (#4) AND (#5) |
| #7 | (#3) AND (#6) |

1. WanFang: (n=8)

| #1 | 题名或关键词:(中医OR TCM OR 中成药 OR 中医药 OR 草药 OR 制剂 OR 方剂 OR 复方) and 题名或关键词:(系统评价 OR meta分析 OR 荟萃分析 OR 系统综述) and 题名或关键词:(高尿酸血症) |
| --- | --- |

1. CNKI: (n=14)

| #1 | (SU=(中医+ TCM + 中成药 + 中医药 + 草药 + 制剂 + 方剂+ 复方) and SU=(系统评价 + meta分析 + 荟萃分析 + 系统综述) and SU=(高尿酸血症)) 资源范围：学术期刊; 中英文扩展; 时间范围：更新时间：不限; 来源类别：全部期刊; |
| --- | --- |

1. VIP: (n=12)

| #1 | (((((题名或关键词=系统评价 OR 题名或关键词=meta分析) OR 题名或关键词=荟萃分析) OR 题名或关键词=系统综述) AND ((((((题名或关键词=中医 OR 题名或关键词=TCM ) OR 题名或关键词=中医药) OR 题名或关键词=复方) OR 题名或关键词=方剂) OR 题名或关键词=制剂) OR 题名或关键词=草药)) AND 题名或关键词=高尿酸血症) |
| --- | --- |

**Supplementary Table S2. Citation Matrix.**

| **Original RCTs** | **SRs included in this study** | | | | | | | | | | |
| --- | --- | --- | --- | --- | --- | --- | --- | --- | --- | --- | --- |
|  | **Wang M,2022** | **Wu FS,2017** | **Liu YJ,2017** | **Qiao LM,2020** | **Xu CX,2025** | **Wu YY,2024** | **Pan Y,2024** | **Huo JJ,2015** | **Shu ZM,2017** | **Wang Y,2022** | **Chen LQ,2020** |
| Bao Y,2014 | 0 | 0 | 0 | 0 | 1 | 0 | 0 | 0 | 0 | 0 | 0 |
| Cai S,2014 | 0 | 0 | 0 | 0 | 0 | 1 | 0 | 0 | 0 | 0 | 0 |
| Cai ZY,2007 | 0 | 0 | 0 | 0 | 1 | 0 | 0 | 0 | 0 | 0 | 0 |
| Chen F,2014 | 0 | 0 | 0 | 0 | 1 | 0 | 0 | 0 | 0 | 0 | 0 |
| Chen JJ,2014 | 1 | 0 | 0 | 0 | 0 | 0 | 0 | 1 | 0 | 0 | 0 |
| Chen Q,2009 | 0 | 0 | 0 | 0 | 0 | 0 | 0 | 0 | 0 | 0 | 1 |
| Chen Q,2021 | 0 | 0 | 0 | 0 | 1 | 0 | 0 | 0 | 0 | 0 | 0 |
| Chen SQ,2022 | 0 | 0 | 0 | 0 | 0 | 1 | 0 | 0 | 0 | 0 | 0 |
| Chen SW,2007 | 1 | 1 | 0 | 0 | 0 | 0 | 0 | 0 | 0 | 0 | 0 |
| Chen WP,2013 | 1 | 0 | 0 | 0 | 0 | 0 | 0 | 0 | 1 | 0 | 0 |
| Chen ZY,2019 | 1 | 0 | 0 | 0 | 0 | 0 | 0 | 0 | 0 | 0 | 0 |
| Deng YF,2020 | 0 | 0 | 0 | 0 | 0 | 0 | 1 | 0 | 0 | 0 | 0 |
| Deng YX,2019 | 0 | 0 | 0 | 0 | 0 | 1 | 1 | 0 | 0 | 0 | 0 |
| Dong DD,2018 | 0 | 0 | 0 | 0 | 1 | 0 | 0 | 0 | 0 | 0 | 0 |
| Gong Y,2006 | 0 | 0 | 0 | 0 | 1 | 0 | 0 | 0 | 0 | 0 | 0 |
| Hong Q,2011 | 0 | 0 | 1 | 1 | 0 | 0 | 0 | 0 | 0 | 0 | 0 |
| Huang BY,2022 | 0 | 0 | 0 | 0 | 0 | 1 | 0 | 0 | 0 | 0 | 0 |
| Jia JJ,2010 | 0 | 0 | 1 | 1 | 0 | 0 | 0 | 0 | 0 | 0 | 0 |
| Lai Y,2018 | 0 | 0 | 0 | 0 | 1 | 0 | 0 | 0 | 0 | 0 | 0 |
| Leng HC,2021 | 0 | 0 | 0 | 0 | 0 | 0 | 1 | 0 | 0 | 0 | 0 |
| Li HY,2007 | 1 | 0 | 0 | 0 | 0 | 0 | 0 | 0 | 0 | 0 | 0 |
| Li J,2010 | 1 | 1 | 0 | 0 | 0 | 0 | 0 | 1 | 1 | 0 | 0 |
| Li XQ,2011 | 0 | 0 | 1 | 0 | 0 | 0 | 0 | 1 | 0 | 0 | 0 |
| Li Y,2011 | 1 | 0 | 0 | 0 | 0 | 0 | 0 | 0 | 0 | 0 | 0 |
| Li YW,2019 | 1 | 0 | 0 | 0 | 0 | 0 | 0 | 0 | 0 | 0 | 0 |
| Liang H,2014 | 0 | 0 | 0 | 0 | 0 | 0 | 0 | 0 | 0 | 1 | 0 |
| Liang M,2019 | 1 | 0 | 0 | 0 | 0 | 0 | 0 | 0 | 0 | 0 | 0 |
| Liu GX,2011 | 1 | 1 | 0 | 0 | 0 | 0 | 0 | 0 | 0 | 0 | 0 |
| Liu MY,2011 | 0 | 0 | 1 | 1 | 0 | 0 | 0 | 0 | 0 | 0 | 0 |
| Liu W,2016 | 0 | 0 | 1 | 1 | 0 | 0 | 0 | 0 | 0 | 0 | 0 |
| Lu C,2000 | 0 | 0 | 0 | 0 | 0 | 0 | 0 | 0 | 0 | 1 | 0 |
| Lu YP,2016 | 0 | 0 | 0 | 0 | 0 | 0 | 1 | 0 | 0 | 0 | 0 |
| Luo YY,2019 | 0 | 0 | 0 | 0 | 1 | 0 | 0 | 0 | 0 | 0 | 0 |
| Lyu CS,2017 | 1 | 0 | 0 | 0 | 0 | 0 | 0 | 0 | 0 | 0 | 0 |
| Pan QH,2011 | 0 | 1 | 0 | 0 | 0 | 0 | 0 | 0 | 1 | 0 | 0 |
| Pan YZ,2020 | 0 | 0 | 0 | 0 | 0 | 0 | 0 | 0 | 0 | 1 | 0 |
| Qian YZ,2013 | 1 | 1 | 0 | 0 | 0 | 1 | 0 | 0 | 0 | 0 | 0 |
| Rozza,2016 | 0 | 0 | 0 | 0 | 0 | 0 | 0 | 0 | 0 | 0 | 1 |
| San LH,2013 | 0 | 0 | 0 | 0 | 0 | 0 | 0 | 0 | 1 | 0 | 0 |
| Shao P,2009 | 1 | 0 | 0 | 0 | 0 | 0 | 0 | 0 | 0 | 0 | 0 |
| Shi BG,2010 | 1 | 0 | 0 | 0 | 0 | 0 | 0 | 0 | 1 | 0 | 0 |
| Sun H,2016 | 0 | 0 | 1 | 0 | 0 | 1 | 0 | 0 | 0 | 0 | 0 |
| Sun SF,2012 | 1 | 0 | 1 | 1 | 0 | 0 | 0 | 0 | 0 | 0 | 0 |
| Sun WF,2003 | 1 | 0 | 0 | 0 | 0 | 0 | 0 | 0 | 1 | 0 | 0 |
| Tan N,2012 | 0 | 0 | 0 | 1 | 0 | 0 | 0 | 0 | 1 | 0 | 0 |
| Tan N,2013 | 0 | 0 | 1 | 0 | 0 | 0 | 0 | 0 | 0 | 0 | 0 |
| Tang Y,2017 | 0 | 0 | 0 | 0 | 0 | 0 | 1 | 0 | 0 | 0 | 0 |
| Tian WW,2016 | 0 | 0 | 0 | 0 | 0 | 1 | 0 | 0 | 0 | 0 | 0 |
| Wang G,2014 | 0 | 0 | 1 | 1 | 0 | 0 | 0 | 0 | 0 | 0 | 0 |
| Wang HX,2018 | 1 | 0 | 0 | 0 | 0 | 0 | 0 | 0 | 0 | 0 | 0 |
| Wang LY,2006 | 0 | 0 | 0 | 0 | 0 | 0 | 0 | 1 | 0 | 0 | 0 |
| Wang T,2012 | 0 | 0 | 0 | 0 | 0 | 0 | 0 | 1 | 0 | 0 | 0 |
| Wang T,2021 | 0 | 0 | 0 | 0 | 0 | 0 | 1 | 0 | 0 | 0 | 0 |
| Wang Y,2014 | 0 | 0 | 0 | 0 | 0 | 0 | 0 | 0 | 0 | 0 | 1 |
| Wang Y,2019 | 0 | 0 | 0 | 0 | 0 | 0 | 0 | 0 | 0 | 0 | 1 |
| Wang YH,2019 | 0 | 0 | 0 | 0 | 0 | 0 | 0 | 0 | 0 | 1 | 0 |
| Wu N,2020 | 1 | 0 | 0 | 0 | 0 | 1 | 0 | 0 | 0 | 0 | 0 |
| Xiang SW,2009 | 0 | 0 | 0 | 0 | 0 | 0 | 0 | 0 | 0 | 0 | 1 |
| Xie Z,2017 | 0 | 0 | 0 | 0 | 0 | 0 | 0 | 0 | 0 | 0 | 1 |
| Xu H,2013 | 0 | 1 | 1 | 0 | 0 | 0 | 0 | 0 | 0 | 0 | 0 |
| Xu P,2016 | 0 | 0 | 0 | 0 | 0 | 0 | 1 | 0 | 0 | 0 | 0 |
| Xue S,2010 | 0 | 0 | 1 | 1 | 0 | 0 | 0 | 0 | 0 | 0 | 0 |
| Yang GC,2013 | 1 | 0 | 0 | 0 | 0 | 0 | 0 | 0 | 0 | 0 | 0 |
| Yang JY,2020 | 0 | 0 | 0 | 0 | 1 | 0 | 1 | 0 | 0 | 0 | 0 |
| Yang QP,2016 | 0 | 0 | 0 | 0 | 1 | 0 | 0 | 0 | 0 | 0 | 0 |
| Yang W,2012 | 1 | 0 | 0 | 0 | 0 | 0 | 0 | 1 | 1 | 0 | 0 |
| Yang W,2013 | 1 | 0 | 0 | 0 | 0 | 0 | 0 | 0 | 0 | 0 | 0 |
| Yang XF,2015 | 1 | 0 | 0 | 0 | 0 | 0 | 0 | 0 | 0 | 0 | 0 |
| Ye SA,2017 | 1 | 0 | 0 | 0 | 0 | 0 | 0 | 0 | 0 | 0 | 0 |
| Yin DM,2012 | 0 | 1 | 0 | 0 | 0 | 0 | 0 | 1 | 1 | 0 | 0 |
| Yin YH,2008 | 1 | 0 | 0 | 0 | 0 | 0 | 0 | 0 | 0 | 0 | 0 |
| Yu JW,2013 | 1 | 0 | 0 | 0 | 0 | 0 | 0 | 0 | 0 | 0 | 0 |
| Yu L,2009 | 1 | 0 | 0 | 0 | 0 | 0 | 0 | 0 | 0 | 0 | 0 |
| Yu M,2019 | 1 | 0 | 0 | 0 | 0 | 0 | 0 | 0 | 0 | 0 | 0 |
| Yu XN,2018 | 0 | 0 | 0 | 0 | 0 | 0 | 0 | 0 | 0 | 0 | 1 |
| Zeng BS,2020 | 0 | 0 | 0 | 0 | 0 | 0 | 1 | 0 | 0 | 0 | 0 |
| Zhang DN,2021 | 0 | 0 | 0 | 0 | 0 | 0 | 1 | 0 | 0 | 0 | 0 |
| Zhang FR,2006 | 1 | 0 | 0 | 0 | 0 | 0 | 0 | 0 | 0 | 0 | 0 |
| Zhang LL,2005 | 0 | 0 | 0 | 0 | 0 | 0 | 0 | 0 | 0 | 1 | 0 |
| Zhang LQ,2011 | 0 | 0 | 1 | 1 | 0 | 0 | 0 | 1 | 0 | 0 | 0 |
| Zhang M,2008 | 0 | 0 | 0 | 0 | 0 | 0 | 0 | 0 | 1 | 0 | 0 |
| Zhang M,2009 | 0 | 0 | 0 | 0 | 0 | 0 | 0 | 0 | 0 | 0 | 1 |
| Zhang M,2017 | 1 | 0 | 0 | 0 | 0 | 0 | 0 | 0 | 0 | 0 | 0 |
| Zhang PL,2011 | 1 | 0 | 1 | 1 | 0 | 0 | 0 | 1 | 0 | 0 | 0 |
| Zhang XX,2011 | 0 | 1 | 1 | 0 | 0 | 0 | 0 | 0 | 1 | 0 | 1 |
| Zhang XX,2016 | 0 | 0 | 1 | 0 | 0 | 0 | 0 | 0 | 0 | 0 | 0 |
| Zhang ZL,2019 | 1 | 0 | 0 | 0 | 0 | 0 | 0 | 0 | 0 | 0 | 0 |
| Zhao JH,2015 | 0 | 0 | 0 | 0 | 0 | 0 | 1 | 0 | 0 | 0 | 0 |
| Zhao L,2019 | 0 | 0 | 0 | 0 | 1 | 0 | 0 | 0 | 0 | 0 | 0 |
| Zhao MC,2012 | 0 | 1 | 0 | 0 | 0 | 0 | 0 | 0 | 1 | 0 | 0 |
| Zheng G,2012 | 0 | 1 | 0 | 0 | 0 | 0 | 0 | 0 | 0 | 0 | 0 |
| Zhong J,2017 | 0 | 0 | 0 | 0 | 1 | 0 | 0 | 0 | 0 | 0 | 0 |
| Zhong QS,2009 | 0 | 0 | 0 | 0 | 1 | 0 | 0 | 0 | 0 | 0 | 0 |
| Zhong YL,2014 | 1 | 0 | 0 | 0 | 0 | 0 | 0 | 0 | 0 | 0 | 0 |
| Zhou H,2011 | 0 | 0 | 1 | 1 | 0 | 0 | 0 | 0 | 0 | 0 | 0 |
| Zhou HZ,2018 | 1 | 0 | 0 | 0 | 0 | 0 | 0 | 0 | 0 | 0 | 0 |
| Zhou JJ,2010 | 0 | 0 | 0 | 0 | 0 | 0 | 0 | 1 | 1 | 0 | 0 |
| Zhou M,2013 | 0 | 0 | 0 | 0 | 0 | 0 | 0 | 0 | 0 | 0 | 1 |
| Zhou QX,2016 | 1 | 0 | 0 | 0 | 0 | 0 | 0 | 0 | 0 | 0 | 0 |
| Zhu QJ,2009 | 0 | 0 | 0 | 0 | 0 | 0 | 0 | 0 | 1 | 0 | 0 |
| Zhu WP,2008 | 1 | 0 | 0 | 0 | 0 | 0 | 0 | 0 | 0 | 0 | 0 |
| Zou LJ,2015 | 0 | 0 | 0 | 0 | 0 | 0 | 0 | 0 | 0 | 1 | 0 |
| Summary | 35 | 10 | 16 | 11 | 13 | 8 | 11 | 10 | 14 | 6 | 10 |
